# Predicting Ewe Body Condition Score Using Lifetime Liveweight and Liveweight Change, and Previous Body Condition Score Record

**DOI:** 10.3390/ani10071182

**Published:** 2020-07-13

**Authors:** Jimmy Semakula, Rene Anne Corner-Thomas, Stephen Todd Morris, Hugh Thomas Blair, Paul Richard Kenyon

**Affiliations:** 1 School of Agriculture and Environment, Massey University, Private Bag 11222, Palmerston North 4410, New Zealand; R.Corner@massey.ac.nz (R.A.C.-T.); s.t.morris@massey.ac.nz (S.T.M.); h.blair@massey.ac.nz (H.T.B.); p.r.kenyon@massey.ac.nz (P.R.K.); 2 National Agricultural Research Organization, Entebbe P.O. Box 295, Uganda

**Keywords:** accuracy, multivariate, variable, prediction error, record

## Abstract

**Simple Summary:**

This study aimed to investigate the possibility of using lifetime liveweight and liveweight change and previous body condition scores to predict current body condition scores in Romney ewes. Models using a ewe’s lifetime liveweight record alone were poor at predicting ewe body condition scores. A combination of lifetime liveweight, liveweight change, and previous body condition scores improved body condition score prediction. If higher accuracy can be achieved, these prediction equations can be incorporated into the electronic weigh heads of modern weigh systems to automatically give farmers predictions of the body condition score (BCS) of an individual during routine weighing. This would benefit farmers by allowing for targeted nutritional management of individual animals to maximize overall flock productivity.

**Abstract:**

The body condition score (BCS) in sheep (*Ovis aries*) is a widely used subjective measure of body condition. Body condition score and liveweight have been reported to be statistically and often linearly related in ewes. Therefore, it was hypothesized that current BCS could be accurately and indirectly predicted using a ewe’s lifetime liveweight, liveweight change, and previous BCS record. Ewes born between 2011 and 2012 (*n* = 11,798) were followed from 8 months to approximately 67 months of age in New Zealand. Individual ewe data was collected on liveweight and body condition scores at each stage of the annual cycle (pre-breeding, pregnancy diagnosis, pre-lambing, and weaning). Linear regression models were fitted to predict BCS at a given ewe age and stage of the annual cycle using a ewe’s lifetime liveweight records (liveweight alone models). Further, linear models were then fitted using previous BCS and changes in liveweight, in addition to the lifetime liveweight records (combined models). Using the combined models improved (*p* < 0.01) the R^2^ value by 39.8% (from 0.32 to 0.45) and lowered the average prediction error by 10% to 12% (from 0.29 to 0.26 body condition scores). However, a significant portion of the variability in BCS remained unaccounted for (39% to 89%) even in the combined models. The procedures found in this study, therefore, may overestimate or underestimate measures by 0.23 to 0.32 BCS, which could substantially change the status of the ewe, leading to incorrect management decisions. However, the findings do still suggest that there is potential for predicting ewe BCS from liveweight using linear regression if the key variables affecting the relationship between BCS and liveweight are accounted for.

## 1. Introduction

The body condition score (BCS) in sheep (*Ovis aries*) is a widely used subjective measure of the degree of body fatness [1,2,3,4]. It examines the degree of soft tissue coverage (predominantly fat and muscle) in the lumbar region [3,5]. The body condition score utilizes a 0–5 scale using half units or quarter units, and is undertaken by the palpation of the lumbar vertebrae (spinous and transverse process) immediately caudal to the last rib and above the kidneys [5]. Unlike liveweight (LW), BCS is not affected by factors such as variations in gut-fill, fleece weight, pregnancy, and frame size that confound liveweight as a measure of animal size to predict the body condition [5,6]. The body condition score can be easily learned and is cost-effective and requires no specialist equipment [5]. Knowledge of sheep BCS ensures that available feed resources are efficiently utilized, subtle differences in body condition not visibly noticeable are determined, there is instant awareness by producers about major changes in body fatness, and the monitoring of trends in nutrition and body weight.

Even though using BCS offers several advantages over liveweight (LW) to better manage flocks, farmers do not regularly use this technique. For example, while 96% of Australian producers indicated they monitored the body condition, only 7% conducted hands-on BCS [7]. In New Zealand, 4% of farmers [8] used BCS as a management tool. Farmers either rely on a visual inspection, which is inaccurate, or prefer to use liveweight measures only [9]. The reasons for low BCS adoption among farmers include: (1) The subjective nature of BCS, depending on assessor judgement; (2) being labor-intensive; and (3) needing assessor training, which should be recalibrated over time [5]. Strategies used to increase the use of BCS among farmers and its reliability included farmer training workshops and regular recalibration [5]. However, given the apparently low rate of farmer uptake especially in large extensively managed flock systems, these strategies have been unsuccessful, likely due to not directly addressing how to reduce the labor burden with hands-on BCS. Therefore, it could be argued that reliable and accurate alternative automated methods to estimate the body condition score would be advantageous and would improve farmer uptake and the use of BCS. Ideally, any automatic system to be utilized on extensive and intensive sheep farms would be based on a management tool already utilized on farms to reduce workload, and it would be quick and not subjective in nature.

The relationship between BCS and LW is documented in sheep [5,10], with BCS being positively and generally linearly associated with liveweight [5]. This relationship is known to vary by age, stage of the annual production cycle, and breed of animal [10,11,12]. Studies suggest correlations between BCS and LW can be from 0.20 to 0.89 and are stronger in mature ewes (r = 0.73 to 0.89) [12,13]. If the relationship between BCS and LW is predictable, then in theory, measurements of the latter could be used as predictors of BCS. In European sheep breeds, mature liveweight occurs between 25 to 50 months of age [14,15]. Therefore, it could be postulated that, at approximately three years of age, when mature liveweight is reached, a stable base BCS–LW relationship would be established. If this was indeed the case then, as a sheep ages further, future liveweights, based on body condition score-liveweight prediction equations, could be used to predict a BCS or change in BCS with a fair degree of accuracy and reduce the need for hands-on BCS measurement.

In large extensive flock systems, farmers regularly weigh sheep and are increasingly using electronic tags [8]. Both conventional and modern weighing systems combined with individual electronic identification can now allow lifetime data to be collected more easily and quickly on large sheep flocks. Using this technology, combined with an individual BCS at a given point in their lifetime can, therefore, allow a specific stage of life BCS liveweight relationship to be developed. Thus, using a set of established equations it should be possible to have a predicted BCS instantly calculated at each live weighing for each sheep. However, these methods have yet not been developed. If these could be developed, they could be incorporated into the electronic weigh heads of modern weigh systems to give farmers predictions of BCS. To date, this has not been tested. The aim of this study was to investigate the possibility of using lifetime liveweight, liveweight change, and previous BCS to predict a ewe’s current body condition score.

## 2. Materials and Methods

### 2.1. Farms and Animals Used and Data Collection

The current study utilized data collected between 2011 and 2016 from two commercial New Zealand sheep farms (A and B) as part of normal routine farm management. All ewes (Romney breed) were weighed (to the nearest 0.1 kg) using static digital weighing scales (Tru-Test group, model XR5000). Body condition scores were undertaken by experienced assessors using a 1–5 scale (1 = thin, 5 = obese) with sheep assessed to the nearest 0.5 of a BCS [3,5] at four time periods within an annual cycle, namely, pre-breeding, pregnancy diagnosis, pre-lambing, and weaning. Data were collected over six individual years as ewes aged 8–18 to ≥67 months. Table 1 gives a chronological summary of the measurements/variables used in the study. The sample sizes as at mating for each year of study were 11,798, 8393, 6651, 5149, 4314, and 1437 ewes (Table 2 and Table 3). A full description of the data used in the present study and sample characteristics is given in [12]. In their study, Semakula and Corner–Thomas [12] determined the nature of the association of the relationship between LW and BCS and the factors affecting this relationship. The present study explores the possibility of utilizing the established relationship in the study above to indirectly predict a ewe’s current BCS using previous liveweight, liveweight change, and BCS record.

### 2.2. Statistical Analyses

Data were analyzed using R program version 3.3.4 [16] with package extensions in the caret package [17]. It was not possible to observe a strict measurement collection protocol; therefore, missing values occurred in our dataset. To fill in the missing values, we used the pre-Process function from the caret package in R (bagimput method). This method constructs a “bagging” model for each of the available variables based on regression trees, using all other variables as predictors while preserving the original data distribution structure [17]. Live weight data were also normalized and centered during analysis using the same pre-Process function above.

The body condition score data is both discrete and ordered in nature, which makes multi-class classification regression approaches such as ordinal logistic or nominal regression more suitable for its analysis. However, when the underlying assumptions are grossly violated or when classes are extremely imbalanced [18], classification statistical methods become less accurate [19]. Triguero and del Río [20] categorize class imbalances above 50:1 for any two outcomes as high-class imbalance. Strategies to overcome the challenge of class imbalance include oversampling, under-sampling, and synthetic minority oversampling [21]. Such methods of circumventing class imbalances hold in cases of “reasonable” imbalance [20]. In the case of high-class imbalance, the samples generated become less representative of the true sample distribution leading to under or overfitting the model. In the present study, it was not possible to conduct classification regression using a full BCS scale (1–5) due to high-class imbalance (1:1 to 1:280). The mitigation approaches to high-class imbalance may include modification of scale to a size that improves the distribution of values (not favorable for full-scale prediction) or the use of other statistical methods robust to class imbalance such as multivariate (multiple regression) methods for interval and continuous data [22]. In cattle, multivariate regression has successfully been used to predict BCS from physical body measurements and 3D camera image data [23]. Therefore, based on the previously outlined points, multivariate linear regression was used to predict ewe BCS from liveweight.

### 2.3. Variable Selection, Model Building, and Validation

Initially, the best predictor combinations for each BCS were selected through the regularization and variable selection technique implemented in the R program [16] using the elastic net method in the glmnet extension [24] in the caret package [17]. The elastic net method combines the power of two penalized-regularization methods (ridge and lasso regression) to search for the number of variables as well as handling collinearity [25].

All models were constructed, fitted, and validated using algorithms, implemented in four steps. The steps included; (i) data partitioning, (ii) resampling, (iii) model training, and (iv) validation. Data partitioning involved dividing the initial dataset (with stratification preserving the class proportions) into training and testing datasets in a ratio of 3:1, with replacement. Resampling involved using bootstrapping and aggregation [26] procedures implemented in R [16] using the caret package [17] to select 10 subsamples from the training set and repeating the resampling three times. Model training involved fitting of the model using the training dataset subsamples (10) from which nine were used for computing the parameters (i.e., β) while the remaining one part was used for error estimation (ε). Finally, all parameter estimates or probabilities from each subsample were averaged to get the final value (estimate) with a 95% confidence interval.

Two multiple regression approaches were evaluated for the possibility of predicting BCS on a full scale, namely, the general linear model (LM) using the generalized least squares (GLS) and linear mixed-effects model (LMM) procedure in the nlme package [27]. The LM was selected for subsequent analysis; the variance between LMM and GLS showed no significant difference (*p* < 0.05). Using the selected best predictors for each BCS, LM regression equations were fitted to predict the current BCS using lifetime (present and previous) liveweight records (liveweight alone models). Later, the models were modified by using previous liveweight change and BCSs in addition to lifetime liveweight (combined models). Consequently, forty-eight (48) regression equations were generated for BCS prediction, half of which were from using liveweight alone models and the remaining half from the combined models. Lifetime measurements refer to those ewe measurements taken at the same and previous time points, whereas previous measurements only refer to those preceding the current one. Liveweight change was defined as sequential retrospective change in liveweight between individual time points.

### 2.4. Model Performance Evaluation

The calibration model performance (based on training dataset) was assessed using two metrics [28,29]: The adjusted coefficient of determination (adj. R^2^: accounting for number of predictors) and the root mean square error (RMSE). The validation for each BCS prediction model was conducted using the testing dataset, each repeated 1000 times. Several metrics were considered when assessing the quality of models, including the coefficient of determination (R^2^ or unadjusted R^2^), bias, root mean squared error (RMSE), residual prediction deviation (RPD), and the ratio of performance to interquartile distance (RPIQ) [30]. The success of the predictions for individual samples was determined using the mean absolute percent error (MAPE) and the relative prediction error percent (RPE). The best model would have the highest R^2^, RPD, and RPIQ, and the lowest RMSE, MAPE and RPE. In addition, RPD was classified [31] into three different categories: Weak prediction (RPD < 1.4), reasonable (1.4 < RPD < 2.0), and excellent (RPD > 2.0). In a similar manner [32], RPIQ was divided into four categories: Very poor prediction (RPIQ < 1.4), fair (1.4 < RPIQ < 1.7), good (1.7 < RPIQ < 2.0), very good (2.0 < RPIQ < 2.5), and excellent (RPIQ > 2.5).

## 3. Results

### 3.1. Correlation between all BCS and Liveweights

There was an association between liveweight and BCS in all age groups and stages of the annual cycle, but the association was characterized as being weak to moderate (Table 2a,b). The relationships, however, were stronger when liveweight and BCS measurements were from the same time point (0.25 ≤ r ≤ 0.67), compared to when lifetime (i.e., including the same time point and previous) records were used (−0.18 ≤ r ≤ 0.67). In terms of the stage of the annual cycle, the correlation was strongest at weaning (−0.08 ≤ r ≤ 0.67) and weakest pre-lambing (−0.18 ≤ r ≤ 0.49). Full descriptive statistics of the data used in this study can be found in Semakula and Corner–Thomas [12].

### 3.2. Linear Regression (Prediction of BCS)

#### 3.2.1. Coefficient of Determination (R^2^) and Number of Predictors

**Figure 1:**
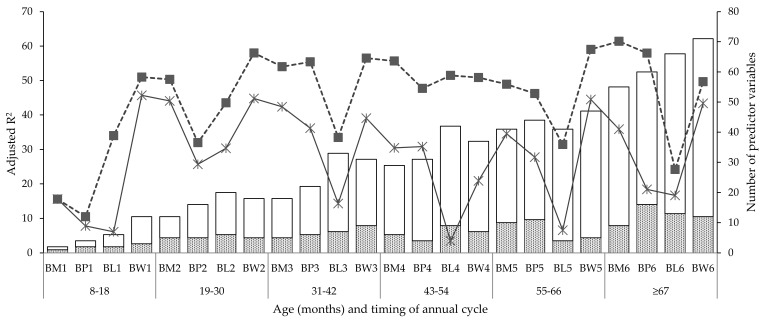
Adjusted R^2^ (solid line: Liveweight alone models, dashed: Combined models) and number of predictors (dotted bar: Liveweight alone models, white bar: Combined models) for BCS prediction across the stage of the annual production cycle and ewe age group. BM, BP, BL, BW indicate body condition score prior to pre-breeding, at pregnancy diagnosis, prior to lambing, and at weaning, respectively.

To predict current BCS, all current and previous individual liveweights (liveweight alone models) were included in linear regression equations. Across age groups, the change in adjusted R^2^ (based on training dataset) value showed no clear pattern (Figure 1). The adjusted R^2^ values averaged 0.32 and did not get above 0.49, regardless of the time point. There was no trend for adjusted R^2^ to improve at older ages, when a greater amount of previous liveweight information was known. It was observed that, in general, the adjusted R^2^ value was highest at weaning but lowest at pre-lambing.

The average number of liveweight predictors (significant variables) for BCS prediction was seven (1 to 16), with no clear pattern of change over time (Table A1 and Table A2). To improve the prediction of current BCS, a combination of all preceding BCS and prior liveweights and their sequential retrospective differences (change in liveweight between individual time points) were included in the regression equations (combined models) and are shown in Table A3 and Table A4. The number of significant predictors for BCS was higher (average: 25, from 1 to 59) in the combined models compared to liveweight alone models.

The adjusted R^2^ values (Figure 1) for ewe BCS prediction ranged from 0.11 to 0.61 for liveweight alone models or combined models. Although there was no clear trend for adjusted R^2^ improvement with age, it appeared to be affected by stage of the annual cycle. Notable was the generally low adjusted R^2^ value at pre-lambing in both combined and liveweight alone models. The adjusted R^2^ increased with the number of variables in the combined model in a similar manner to the liveweight alone models. Using more predictors in addition to liveweight increased the adjusted R^2^ value by 39.8% (from 32.5 to 45.4%) or 1.4 times, and the number of significant predictors at each stage of the annual cycle by 3.6 (average number of variables for combined models liveweight alone models divided by the average number of variables for liveweight alone models) times. A significant portion of the variability in BCS remained unaccounted for (38–89%) in the combined models, with some of the initial liveweight variables in the liveweight alone models being considered non-significant (*p* > 0.05) in the combined models.

#### 3.2.2. Prediction Error Metrics

The BCS model prediction error metrics (MAE, RMSE, MAPE, RMSPE) varied across age group and stage of the annual production cycle when liveweight or combined models were used to predict BCS (Table 4 and Table 5). The average prediction errors associated with BCS prediction in liveweight and in the combined models in terms of MAE and RMSE were 0.26 (0.23 to 0.32) and 0.32 (0.28 to 0.41) body condition scores, respectively. The magnitude of the error values was categorized as being moderate to high in both the liveweight and combined models, given the scale of measurement and smallest unit of measurement (0.5). The BCS predictions using the liveweight alone models were, on average, 9.3 (7.60% to 11.50%) to 11.6 (9.50% to 14.62%) from the actual value. The models were categorized as weak (RPD: 1.02 to 1.39) or very poor to fair (RPIQ: 1.28 to 1.79).

The model prediction error metrics for the combined models varied across age group and stage of the annual production cycle but were significantly (*p* < 0.01) reduced compared to the liveweight alone models. The average prediction error associated with BCS prediction using the combined models in terms of MAE and RMSE was reduced by 0.04 (10% to 12%) body condition scores. Overall the combined models improved BCS prediction from weak to reasonable (PRD: 1.40) or good (RPID: 1.75). The results also showed positive and negative biases for both models, and an indication of the tendency to underestimate or overestimate BCS measurement.

## 4. Discussion

The aim of this study was to explore the possibility of predicting BCS from lifetime liveweight, liveweight change, and previous BCS over time in ewes as they aged from eight through to approximately sixty-seven months. This appears to be the first study to attempt this in sheep. Previous studies have examined the relationship between liveweight and BCS at a given time point [10,11,12].

This study demonstrated that despite BCS and LW being linearly correlated [5,12], the relationship was weak when predicting using linear regression, even in older individuals which would have attained maturity. The results also indicated that the role of prior liveweight measurements in predicting BCS diminished as the time gap between measurement points increased. This indicates that using early life liveweights alone would likely be unreliable in predicting future BCS. Further, the effect of liveweight change on BCS prediction was more significant during the early years of a ewe than in her later years, which implies that liveweight change may cease to be an important predictor of BCS after maturity is reached.

The variability in BCS explained for both liveweight and combined models increased with the number of predictors in the model. This was expected as it is known that as the number of predictors that significantly relate to the dependent variable increase, the proportion of the variance due to the regression increases [33]. However, in this study, a considerable amount of variability in BCS (0.58 ≤ R^2^ ≤ 0.91 and 0.39 ≤ R^2^ ≤ 0.89) remained unaccounted for in both liveweight alone and combined models, respectively. Potential reasons for the apparent failure for both liveweight alone and combined models to account for more of the variability in BCS include BCS binning (due to not being a continuous variable), assessor consistency over time, losses in liveweight due to gut-fill and urination when ewes are weighed at different times, fleece weight and wetness, and confounding of liveweight with conceptus weight. The consistency of the BCS data can vary between 5% to 27% and 40% to 60%, and within 16% to 44% and 60% to 90% for inexperienced and experienced assessors, respectively [5,34]. Liveweight losses resulting from fluctuations in the gut-fill can account for between 5% and 23% of total liveweight in ruminants [35,36]. Thus, when an individual’s liveweight was recorded with respect to when the animal was fed could influence the accuracy of a liveweight. The present study did not measure for individual time off feed prior to weighing, a function that many electronic weighing systems have the potential to do. As the pregnancy advances, conceptus weight increases depending on the number of fetuses carried [37], which could have affected the liveweight and BCS differently. The present dataset did not have information on the individual stage of pregnancy for each ewe. Future studies should examine if the accuracy of the prediction can be ameliorated by incorporating these two variables. In regression, the independent variable measurement is assumed to be measured with high precision; thus, it is not expected to contribute to residual error [38]. Therefore, losses in liveweight due to gut-fill changes and urination in relation to when ewes are weighed at different times and the effect of pregnancy on liveweight are of concern, as they affect liveweight, which is an independent variable for BCS prediction. When independent variables are not exact, estimations based on the standard assumption lead to inconsistent parameter estimates even in very large samples [39,40]. Thus, if errors in the measurements of liveweight could be minimized, then the resulting error term in the regression could all be attributed to BCS measurement, which should improve the model goodness-of-fit and accuracy. In order to reduce this measurement error, it would be imperative that liveweight losses due to delayed weighing be accounted for with respect to time of delay (the period from when the animal last fed to weight recording) in using prediction equations. Time-dependent, liveweight adjusting equations for ewes have been developed but are not regularly used [41].

In the present study, the prediction models using liveweight alone had large error (MAE and RMSE) and low RPD and RPIQ values, which led to high error rates. Combined models reduced the magnitude of all the prediction error metrics to near acceptable levels. Although an error (MAPE or RPE) up to 20% is acceptable for setting dosage rates in the veterinary pharmaceutical industry [42], an error of more than 10% can be problematic [43,44,45] in other agricultural fields. In this study, values were approximately 9% to 12% for liveweight alone models and 8% to 10% for the combined models. The moderate to large error values (one-half to two-thirds of the smallest unit on a 0.5 decimal scale) in BCS prediction in the present study (where a 0.5-unit change in BCS changes the performance rank of a ewe) could greatly influence management decisions. In theory, both models should have had resolutions of approximately 0.02 (maximum span = 0.5/smallest possible increment = (2) ^ maximum range of possible values) body condition score. However, due to the rigid nature (discrete or noncontinuous scale with no values in between the fixed points) of the scale used, such resolutions were not achievable. It has been suggested that decisions concerning targeted feeding and management of ewes to maximum performance were based on a “minima” BCS (i.e., 2.5) or a critical range of BCS values (i.e., 2.5 to 3.5) [5]. The predictions found in this study may, therefore, overestimate or underestimate measures by 0.23 to 0.32 BCS, which could substantially change the status of the ewe, leading to incorrect management decisions, which in turn could reduce flock productivity.

## 5. Conclusions

The combined models improved the proportion of variability in BCS accounted for, as well as the accuracy metrics across age groups and stages of the annual cycle and over time (years), compared to the liveweight alone models. This indicates that BCS could be better predicted if additional variables (liveweight, liveweight change, and previous BCS) were included in the multiple regression equation, rather than lifetime liveweight alone. These relationships could potentially be incorporated in electronic weighing systems that utilize lifetime data. This would be especially useful when applied to large extensively run sheep flocks. However, a significant portion of the variability in BCS remained unaccounted for (39% to 89%) even in the combined models. It is possible that the prediction models could be improved if additional information such as stage of pregnancy, number of fetuses carried, and time off feed was utilized and warrants further investigation.

## Figures and Tables

**Table 1:** Explanation of liveweight, liveweight change, and body condition score (BCS) variables by ewe age group and stage of the annual cycle.

Age (Months)	Stage of the Annual Cycle	* Liveweight	§ BCS	£ Change in Liveweight
8–18	Pre-breeding	WM1	BM1	
	Pregnancy diagnosis	WP1	BP1	WT11(WP1–WM1)
	Pre-lambing	WL1	BL1	WT12(WL1–WP1)
	Weaning	WW1	BW1	WT13(WW1–WL1)
19–30	Pre-breeding	WM2	BM2	T2-T1(VM2–WW1)
	Pregnancy diagnosis	WP2	BP2	WT21(WP2–WM2)
	Pre-lambing	WL2	BL2	WT22(WL2–WP2)
	Weaning	WW2	BW2	WT23(WW2–WL2)
31–42	Pre-breeding	WM3	BM3	T3-T2(VM3–WW2)
	Pregnancy diagnosis	WP3	BP3	WT31(WP3–WM3)
	Pre-lambing	WL3	BL3	WT32(WL3–WP3)
	Weaning	WW3	BW3	WT33(WW3–WL3)
43–54	Pre-breeding	WM4	BM4	T4-T3VM4–WW3
	Pregnancy diagnosis	WP4	BP4	WT41WP4–WM4
	Pre-lambing	WL4	BL4	WT42WL4–WP4
	Weaning	WW4	BW4	WT43WW4–WL4
55–65	Pre-breeding	WM5	BM5	T5-T4(VM5–WW4)
	Pregnancy diagnosis	WP5	BP5	WT51(x2013)
	Pre-lambing	WL5	BL5	WT52(x2013)
	Weaning	WW5	BW5	WT53(WW5−WL5)
≥67	Pre-breeding	WM6	BM6	T6-T5(VM6–WW4)
	Pregnancy diagnosis	WP6	BP6	WT61(WP6–WM6)
	Pre-lambing	WL6	BL6	WT62(WL6–WP6)
	Weaning	WW6	BW6	WT63(WW6–WL6)

* Liveweight; at pre-breeding (WM), pregnancy diagnosis (WP), pre-lambing (WL), and weaning (WW). § BCS; at pre-breeding (BM), pregnancy diagnosis (BP), pre-lambing (BL), and weaning (BW). £ Change in liveweight: WT; change in liveweight between successive measurements within age groups, DT-T; change in liveweight between successive measurements between age groups.

**Table 2:** Correlation coefficients between individual liveweight and body condition scores across stages of the annual production cycle in ewes between 8 and 42 months.

Weight	*n*	Body Condition Score											
		BM1	BP1	BL1	BW1	BM2	BP2	BL2	BW2	BM3	BP3	BL3	BW3
WM1	11,798	0.38	0.13	0.13	−0.05 ^ns^	0.00 ^ns^	0.08	−0.12	0.18	0.02 ^ns^	0.09	0.01 ^ns^	0.19
WP1	11,124	0.32	0.36	0.46	0.11	0.00 ^ns^	0.10	−0.02 ^ns^	0.16	0.05	0.08	0.03 ^ns^	0.22
WL1	8074	0.28	0.18	0.49	0.25	−0.11	0.16	0.43	0.21	0.21	0.18	0.08	−0.04
WW1	8499	0.09	0.25	0.44	0.67	0.41	0.28	0.33	0.12	0.17	0.11	0.06	0.04
WM2	8393	0.12	0.25	0.33	0.54	0.49	0.25	0.26	0.15	0.16	0.14	0.09	0.11
WP2	7991	0.14	0.36	0.29	0.25	0.37	0.39	0.01 ^ns^	0.15	0.04	0.11	0.14	0.30
WL2	5362	0.15	0.34	0.45	0.41	0.29	0.40	0.25	0.11	0.10	0.14	0.07	0.15
WW2	6950	0.13	0.28	0.33	0.25	0.19	0.21	0.11	0.53	0.39	0.32	0.26	0.29
WM3	6651	0.14	0.06	0.12	0.20	0.16	0.24	0.21	0.48	0.51	0.45	0.29	0.21
WP3	6308	0.16	0.13	0.29	0.26	0.13	0.31	0.29	0.46	0.43	0.51	0.32	0.19
WL3	2700	0.13	0.17	0.15	0.20	0.13	0.25	0.24	0.33	0.38	0.45	0.32	0.16
WW3	5579	0.12	−0.03 ^ns^	0.01 ^ns^	0.09	0.12	0.21	0.10	0.38	0.23	0.32	0.26	0.60
WM4	5149	0.12	−0.04	0.02 ^ns^	0.11	0.12	0.22	0.16	0.32	0.24	0.32	0.24	0.43
WP4	4944	0.14	−0.11	0.01 ^ns^	0.13	0.08	0.27	0.30	0.34	0.27	0.39	0.27	0.34
WL4	3224	0.12	−0.03 ^ns^	0.02 ^ns^	−0.03 ^ns^	0.09	0.22	0.13	0.34	0.18	0.31	0.19	0.37
WW4	4440	0.06	0.06	0.06	0.17	0.11	0.13	0.09	0.19	0.17	0.18	0.15	0.21
WM5	4314	0.07	−0.03 ^ns^	−0.02 ^ns^	0.11	0.06	0.14	0.15	0.21	0.19	0.22	0.18	0.15
WP5	4146	0.09	−0.07	0.01 ^ns^	0.16	0.05	0.16	0.25	0.20	0.20	0.25	0.18	0.11
WL5	2677	0.10	−0.11	0.02 ^ns^	0.19	0.02 ^ns^	0.15	0.21	0.16	0.20	0.20	0.08	0.03
WW5	2695	0.08	−0.15	0.01 ^ns^	0.15	0.03 ^ns^	0.16	0.27	0.23	0.22	0.22	0.08	0.08
WM6	1437	0.09	−0.15	−0.06	0.12	−0.02 ^ns^	0.13	0.23	0.16	0.16	0.22	0.10	0.06
WP6	1334	0.09	−0.12	−0.05	0.13	−0.04	0.15	0.28	0.15	0.16	0.23	0.10	0.01 ^ns^
WL6	879	0.08	0.09	0.02 ^ns^	0.11	0.01 ^ns^	0.02 ^ns^	0.05	0.08	0.08	0.15	0.09	0.11
WW6	563	0.06	−0.03 ^ns^	−0.03 ^ns^	0.11	−0.03 ^ns^	0.01 ^ns^	0.05	0.10	0.09	0.08	0.09	0.09

BM, BP, BL, BW indicate body condition score prior to pre-breeding, at pregnancy diagnosis, prior to lambing, and at weaning, respectively. WM, WP, WL, WW indicate liveweight prior to pre-breeding, at pregnancy diagnosis, prior to lambing, and at weaning, respectively. Gray shade (major diagonal) indicates liveweights and BCS correlation coefficient values from the same time point. *n* indicates sample size. ns superscript indicates no significance at *p* < 0.05.

**Table 3:** Correlation coefficients between individual liveweight and body condition scores across stages of the annual production cycle in ewes above 42 months of age.

Weight	*n*	Body Condition Score											
		BM4	BP4	BL4	BW4	BM5	BP5	BL5	BW5	BM6	BP6	BL6	BW6
WM1	11,798	0.03 ^ns^	−0.05	0.3	0.11	0.18	0.06 ^ns^	−0.03 ^ns^	−0.03 ^ns^	−0.09	−0.09	−0.03 ^ns^	0.01 ^ns^
WP1	11,124	0.02 ^ns^	−0.05	0.33	0.13	0.19	0.05 ^ns^	−0.04 ^ns^	−0.05	−0.11	−0.10	0.00 ^ns^	0.04
WL1	8074	0.20	0.10	−0.11	−0.04	−0.03 ^ns^	0.15	0.16	0.21	0.35	0.36	0.11	0.14
WW1	8499	0.13	0.09	−0.18	0.04	0.01 ^ns^	0.04 ^ns^	0.10	0.12	0.15	0.11	0.03 ^ns^	0.06
WM2	8393	0.14	0.08	0.01	0.07	0.10	0.07	0.07	0.08	0.09	0.08	0.06	0.08
WP2	7991	0.04	0.00 ^ns^	0.43	0.21	0.29	0.09	−0.04 ^ns^	−0.08	−0.17	−0.15	0.12	0.04
WL2	5362	0.11	0.10	0.01 ^ns^	0.11	0.17	0.09	0.04 ^ns^	0.05	0.06	0.05	0.10	0.10
WW2	6950	0.13	0.06	0.30	0.19	0.25	0.10	0.04 ^ns^	0.03 ^ns^	−0.07	−0.06	0.06	0.09
WM3	6651	0.23	0.11	0.20	0.12	0.20	0.14	0.11	0.13	0.10	0.11	0.11	0.15
WP3	6308	0.25	0.17	0.26	0.13	0.22	0.21	0.15	0.15	0.14	0.18	0.12	0.18
WL3	2700	0.22	0.14	0.09	0.12	0.20	0.14	0.09	0.15	0.10	0.14	0.12	0.15
WW3	5579	0.47	0.29	0.38	0.19	0.27	0.18	0.11	0.14	0.12	0.15	0.06	0.20
WM4	5149	0.53	0.35	0.33	0.17	0.27	0.22	0.16	0.16	0.17	0.16	0.10	0.22
WP4	4944	0.51	0.46	0.33	0.12	0.24	0.29	0.21	0.23	0.27	0.30	0.14	0.21
WL4	3224	0.32	0.17	0.46	0.20	0.32	0.23	0.10	0.11	0.07	0.11	0.07	0.13
WW4	4440	0.26	0.18	0.18	0.55	0.40	0.31	0.23	0.20	0.15	0.16	0.09	0.22
WM5	4314	0.26	0.16	0.19	0.30	0.48	0.39	0.28	0.20	0.25	0.26	0.15	0.29
WP5	4146	0.28	0.22	0.13	0.20	0.32	0.48	0.33	0.26	0.31	0.33	0.17	0.22
WL5	2677	0.24	0.16	0.03 ^ns^	0.12	0.16	0.20	0.31	0.26	0.24	0.25	0.03 ^ns^	0.16
WW5	2695	0.27	0.15	0.05	0.12	0.14	0.24	0.29	0.63	0.45	0.39	0.20	0.25
WM6	1437	0.28	0.15	0.03 ^ns^	0.06	0.14	0.18	0.25	0.38	0.59	0.49	0.24	0.32
WP6	1334	0.24	0.15	0.04	0.03 ^ns^	0.07	0.21	0.19	0.33	0.48	0.56	0.25	0.28
WL6	879	0.17	0.07	0.05	0.15	0.22	0.13	0.09	0.25	0.25	0.34	0.28	0.28
WW6	563	0.16	0.04	0.07	0.13	0.16	0.11	0.01 ^ns^	0.19	0.24	0.26	0.27	0.64

BM, BP, BL, BW indicate body condition score prior to pre-breeding, at pregnancy diagnosis, prior to lambing, and at weaning, respectively. WM, WP, WL, WW indicate liveweight prior to pre-breeding, at pregnancy diagnosis, prior to lambing, and at weaning, respectively. Gray shade (major diagonal) indicates liveweights and BCS correlation coefficient values from the same time point. *n*: indicates sample size. ns: superscript indicates no significance at *p* < 0.05.

**Table 4:** Coefficient of determination (unadjusted R^2^), bias, root mean square error (RMSE), mean absolute error percent (MAPE), relative prediction error (RPE) residual prediction deviation (RPD), and the ratio of performance to interquartile distance (RPIQ) based on testing data for the prediction of BCS in ewes between 8 and 42 months by stage of the annual production cycle using liveweight (liveweight alone models) and a combination of predictors (combined models).

	Age Group											
	8–18				19–30				31–42			
	BM1	BP1	BL1	BW1	BM2	BP2	BL2	BW2	BM3	BP3	BL3	BW3
BCS range	1.5–4.5	1.5–4.5	1.5–4.0	1.5–4.5	1.5–5.0	1.5–4.0	1.5–4.0	1.5–5.0	1.5–4.5	1.5–4.0	1.5–4.0	1.0–4.5
	**Liveweight Alone Models (a)**											
R^2^%	15.7	9.1	6.1	45.4	39.4	22.6	26.9	43.7	42.2	24.1	12.4	40.1
Bias	0.01	0.002	−0.01	0.00	0.01	−0.01	0.00	0.00	0.00	0.00	0.01	0.01
MAE	0.30	0.31	0.32	0.27	0.24	0.24	0.25	0.30	0.23	0.24	0.28	0.26
RMSE	0.38	0.43	0.38	0.53	0.27	0.30	0.32	0.38	0.28	0.31	0.35	0.33
MAPE%	11.11	13.15	10.54	9.27	11.06	9.11	9.33	10.78	8.29	8.39	9.77	8.94
RPE%	12.89	14.4	12.36	12.12	11.76	11.39	11.95	13.66	10.09	10.84	12.12	11.35
RPD	1.12	1.06	1.03	1.36	1.20	1.14	1.16	1.22	1.28	1.23	1.09	1.31
RPIQ	1.32	1.28	1.47	1.47	1.52	1.67	1.56	1.32	1.79	1.61	1.43	1.52
	**Combined Models (b)**											
R^2^%	15.7	10.8	35.2	50.0	50.3	34.0	41.2	58.9	53.6	55.5	32.3	56.7
Bias	0.01	0.00	−0.01	−0.01	0.004	0.00	−0.01	−0.01	−0.003	0.00	0.001	−0.01
MAE	0.30	0.02	0.23	0.25	0.22	0.22	0.21	0.24	0.19	0.20	0.31	0.23
RMSE	0.38	0.02	0.28	0.32	0.28	0.28	0.28	0.31	0.24	0.26	0.24	0.29
MAPE%	11.11	2.47	8.35	8.92	7.85	8.36	7.84	8.66	6.849	7.21	8.4	7.926
RPE%	12.89	2.47	10.17	11.41	9.98	10.64	10.45	11.19	8.65	9.37	10.85	9.99
RPD	1.12	1.19	1.23	1.43	1.43	1.23	1.31	1.55	1.47	1.36	1.22	1.51
RPIQ	1.32	1.50	1.78	1.56	1.79	1.79	1.78	1.62	2.08	1.92	1.61	1.72

BM, BP, BL, BW indicate body condition score prior to pre-breeding, at pregnancy diagnosis, prior to lambing, and at weaning, respectively. Interpretation of measures: The best model has the highest R^2^, RPD, and RPIQ, and the lowest RMSE, MAPE and RPE. Ranges for values: R^2^ (0: Indicates that the model explains none of the variability of the response data around its mean, 1.0 indicates that the model explains all the variability). RPD (< 1.4: Weak, 1.4 < RPD < 2.0: Reasonable, > 2.0: Excellent). RPIQ (< 1.4: Very poor, 1.4 < RPIQ < 1.7: Fair, 1.7 < RPIQ < 2.0: Good, 2.0 < RPIQ < 2.5: Very good, > 2.5: Excellent). (**a**) Liveweight alone models indicate all previous and current weights. (**b**) Combined models indicate all previous and current weights, liveweight changes, and previous BCSs. Bias (Positive value indicates overestimation; negative sign indicates underestimation).

**Table 5:** Coefficient of determination (unadjusted R^2^), bias, root mean square error (RMSE), mean absolute error percent (MAPE), relative prediction error (RPE) residual prediction deviation (RPD), and the ratio of performance to interquartile distance (RPIQ) based on testing data for the prediction of BCS in ewes above 42 months of age by stage of the annual using liveweight (liveweight alone models) and a combination of predictors (combined models).

	Age Group											
	43–54				55–66				≥67			
	BM4	BP4	BL4	BW4	BM5	BP5	BL5	BW5	BM6	BP6	BL6	BW6
BC range	1.0–4.0	1.0–4.0	1.5–4	1.5–4.0	1.0–4.0	1.0–4.0	2.0–4.0	1.0–4.0	1.5–4.0	1.5–4.5	1.5–3.5	1.5–4.5
	**Liveweight Alone Models (a)**											
R^2^%	37.5	32.1	15.3	40.2	33.7	25.9	15.1	42.4	34.9	36.2	12.6	41.8
Bias	−0.004	0.01	0.01	0.01	−0.01	−0.01	0	−0.02	0.01	−0.02	−0.01	−0.01
MAE	0.25	0.24	0.24	0.24	0.24	0.24	0.26	0.27	0.24	0.31	0.25	0.27
RMSE	0.31	0.31	0.32	0.31	0.29	0.33	0.32	0.34	0.31	0.38	0.32	0.34
MAPE%	8.28	8.30	8.90	9.05	10.03	8.29	9.21	10.38	7.86	9.80	9.61	9.69
RPE%	10.26	10.71	11.87	11.68	12.67	11.4	11.33	13.03	10.15	14.66	11.75	12.2
RPD	1.27	1.21	1.26	1.30	1.13	1.14	1.02	1.32	1.34	1.13	1.06	1.39
RPIQ	1.61	1.56	1.55	1.61	1.39	1.51	1.56	1.40	1.61	1.32	1.56	1.47
	**Combined Models (b)**											
R^2^%	52.6	51.3	52.3	47.9	52.4	49.5	27.8	58.3	63.2	65.4	33.9	43.0
Bias	−0.003	−0.007	−0.013	0.012	0.002	0.009	−0.014	−0.001	0.011	−0.001	0.004	−0.007
MAE	0.21	0.20	0.22	0.22	0.22	0.20	0.22	0.22	0.20	0.22	0.23	0.30
RMSE	0.26	0.26	0.29	0.28	0.29	0.25	0.28	0.28	0.25	0.27	0.28	0.3756
MAPE%	6.94	6.9	8.19	8.28	8.30	6.89	7.78	8.35	6.53	6.75	8.52	10.68
RPE%	8.59	8.97	10.42	10.55	10.56	8.62	9.89	10.62	8.17	8.28	10.84	13.17
RPD	1.48	1.42	1.47	1.38	1.53	1.42	1.16	1.53	1.61	1.71	1.25	1.31
RPIQ	1.92	1.92	1.79	1.79	1.79	2.00	1.79	1.79	2.00	1.85	1.79	1.35

BM, BP, BL, BW indicate body condition score prior to pre-breeding, at pregnancy diagnosis, prior to lambing, and at weaning, respectively. Interpretation of measures: The best model has the highest R^2^, RPD, and RPIQ, and the lowest RMSE, MAPE and RPE. Ranges for values: R^2^ (0: Indicates that the model explains none of the variability of the response data around its mean, 1.0 indicates that the model explains all the variability). RPD (< 1.4: Weak, 1.4 < RPD < 2.0: Reasonable, > 2.0: Excellent). RPIQ (< 1.4: Very poor, 1.4 < RPIQ < 1.7: Fair, 1.7 < RPIQ < 2.0: Good, 2.0 < RPIQ < 2.5: Very good, > 2.5: Excellent). (**a**) Liveweight alone models indicate all previous and current weights. (**b**) Combined models indicate all previous and current weights, liveweight changes, and previous BCSs. (Positive value indicates overestimation; negative sign indicates underestimation).

## Appendix A

**Table A1:** Linear regression intercepts and coefficients and adjusted R^2^ for the prediction of BCS from liveweight (liveweight alone models) between 8–18 and 32–43 months of ewes age across stages of reproductive cycle.

Predictor	BM1	BP1	BL1	BW1	BM2	BP2	BL2	BW2	BM3	BP3	BL3	BW3
WM1	0.04	−0.02	−0.01	−0.02	−0.01	−0.01	−0.02		−0.01	−0.01	−0.01	−0.01
WP1		0.04	0.02		0.01	−0.03	−0.03	−0.03	−0.01	−0.01		0.01
WL1				−0.01	−0.02	0.02	0.02	0.02				−0.01
WW1				0.05	0.01		0.01	−0.01		−0.01	−0.01	
WM2					0.03	0.01	0.01			−0.01		−0.01
WP2						0.03		−0.01	−0.01		0.01	0.01
WL2							0.01				−0.01	−0.01
WW2								0.05	0.01			
WM3									0.04	0.01	0.01	−0.01
WP3										0.05	0.01	−0.01
WL3											0.02	
WW3												0.05
WM4												
WP4												
WL4												
WW4												
WM5												
WP5												
WL5												
WW5												
WM6												
WP6												
WL6												
WW6												
Intercept	1.40	2.14	2.6	1.27	1.33	1.62	2.26	1.26	1.69	1.26	1.94	1.84
Adjusted R^2^	14.1	8.19	6.2	45.4	38.4	25.5	24.8	36.4	38.7	38	14.9	48.9

BM, BP, BL, BW indicate body condition score prior to pre-breeding, at pregnancy diagnosis, prior to lambing, and at weaning, respectively. WM, WP, WL, WW indicate liveweight prior to pre-breeding, at pregnancy diagnosis, prior to lambing, and at weaning, respectively. Blank space indicates coefficient non-significant at *p* < 0.05. Model example for BCS estimation (e.g., BM1 = 1.41 + 0.04 WM1, adj. R^2^ = 14%).

**Table A2:** Linear regression intercepts and coefficients and adjusted R^2^ for the prediction of ewe BCS from liveweight (liveweight alone models) above 43 months of age across stages of reproductive cycle.

Predictor	BM4	BP4	BL4	BW4	BM5	BP5	BL5	BW5	BM6	BP6	BL6	BW6
WM1	−0.01	−0.01				−0.01	−0.01	−0.01	−0.01	−0.02	−0.01	−0.02
WP1	−0.01	−0.01	0.01		0.01				−0.01	−0.01		
WL1			−0.02	−0.01	−0.01					0.01		0.01
WW1			−0.02	−0.01	−0.01	−0.01				−0.01	−0.01	−0.01
WM2			−0.01	−0.01	−0.01	−0.01		−0.01		0.01		0.01
WP2	−0.01		0.02	0.01	0.01				−0.01		0.01	−0.02
WL2					0.01	0.01						0.01
WW2	−0.01		0.01		0.01					−0.01		
WM3		−0.01	−0.01	−0.01	−0.01	−0.01			−0.01	−0.01	0.01	
WP3						0.01					−0.01	
WL3						−0.01				0.01		
WW3	0.02		0.01			−0.01			−0.01		−0.01	
WM4	0.03									−0.01		
WP4		0.04		−0.01		0.01			0.01	0.02	0.01	
WL4			0.02							−0.01	−0.01	−0.01
WW4				0.04	0.01							
WM5					0.03	0.01	0.01	−0.01			0.01	0.01
WP5						0.03	0.01			0.01	0.01	−0.01
WL5							0.01	−0.01	−0.01	−0.01	−0.02	−0.01
WW5								0.05	0.01	0.01		
WM6									0.04	0.01	0.01	
WP6										0.03		−0.01
WL6											0.02	
WW6												0.06
Intercept	1.59	2.30	2.40	1.72	1.46	1.59	1.92	1.65	1.71	1.60	1.96	1.05
Adjusted R^2^	44.7	32.35	48.9	41.66	36.6	28.01	14.8	52.86	52.59	39.27	11.6	46.94

BM, BP, BL, BW indicate body condition score prior to pre-breeding, at pregnancy diagnosis, prior to lambing, and at weaning, respectively. WM, WP, WL, WW indicate liveweight prior to pre-breeding, at pregnancy diagnosis, prior to lambing, and at weaning, respectively. Blank space indicates coefficient non-significant at *p* < 0.05. Model example for BCS estimation (e.g., BM4 = 1.59–0.01 WM1+ … + 0.03 WM4, adj. R^2^ = 45%).

**Table A3:** Linear regression intercepts and coefficients and adjusted R^2^ for the prediction of ewe BCS from combined models (that included lifetime liveweight, liveweight change, and previous BCS) between 8–18 and 32–43 months of ewe age across stages of reproductive cycle.

Predictor	BM1	BP1	BL1	BW1	BM2	BP2	BL2	BW2	BM3	BP3	BL3	BW3
WM1	0.04			−0.01		−0.01	−0.03		−0.01		−0.01	−0.01
BM1		0.16	0.015	0.016	0.011	0.018	0.09	0.011	0.06	0.08	0.07	0.01
WP1				−0.02		−0.02	0.02			−0.01		
DWT11		0.11	0.01	0.01			−0.02		−0.01			−0.01
BP1			0.04	0.07	0.011	−0.014	−0.04	−0.019	0.08	0.01	0.07	0.013
DWT12			0.01				0.01					−0.01
WL1				0.04		−0.01	−0.01	−0.01			0.01	
BL1				0.01	0.09	0.012	0.04	0.05	0.05	0.01	0.04	0.08
DWT13				0.05	0.02	−0.02	−0.01	−0.01			0.01	
WW1				0.01		0.01	0.02			−0.01	−0.02	
BW1					0.028	0.08	0.05	0.07	0.03	−0.01	0.03	0.02
DT2-T1					0.02							
WM2					0.02				−0.01	−0.01	−0.04	−0.01
BM2						0.013	−0.03	0.08	0.09	0.09	−0.01	0.09
DWT21							−0.01				−0.04	
WP2						0.03			−0.01		0.02	−0.01
BP2							0.051	0.024	0.01	0.013	0.01	0.03
DWT22								0.02			−0.02	−0.02
WL2								0.07		0.01	0.02	0.02
BL2								0.011	0.09	0.07	0.015	−0.07
DWT23								0.09		0.01		
WW2								−0.04		−0.02	−0.02	
BW2									0.023	0.018	0.03	0.04
DT3-T2											−0.01	
WM4									0.03		−0.02	−0.01
BM3										0.022	0.011	0.01
DWT31											−0.03	
WP3										0.04	0.08	−0.02
BP3											0.036	0.06
DWT32											0.04	
WL3											−0.03	
BL3												0.025
DWT33												0.01
WW3												0.05
Intercept	1.40	2.30	1.20	0.83	0.42	1.12	1.90	0.65	0.52	0.10	0.22	0.30
Adjusted R^2^	14.10	10.5	34.0	51.0	50.33	32.0	43.55	58.0	54.02	55.43	33.48	56.52

BM, BP, BL, BW indicate body condition score prior to pre-breeding, at pregnancy diagnosis, prior to lambing, and at weaning, respectively. WM, WP, WL, WW indicate liveweight prior to pre-breeding, at pregnancy diagnosis, prior to lambing, and at weaning, respectively. DWT, DW-T indicate liveweight change within age group and between age groups, respectively. Blank space indicates coefficient non-significant at *p* < 0.05. Model example for BCS estimation (e.g., BM1 = 1.41 + 0.04 WM1, adj. R^2^ = 14%).

**Table A4:** Linear regression intercepts and coefficients and adjusted R^2^ for the prediction of ewe BCS from combined models (that included lifetime liveweight, liveweight change, and previous BCS) above 43 months of ewe age across stages of reproductive cycle.

Predictor	BM4	BP4	BL4	BW4	BM5	BP5	BL5	BW5	BM6	BP6	BL6	BW6
WM1			0.01		0.01				0.02			−0.02
BM1	0.06	0.01		0.03	−0.01	0.03	0.02	−0.03	0.02	0.04	0.03	0.07
WP1	−0.01	−0.01							−0.02		−0.01	
DWT11	0.01								0.01	0.01		
BP1	0.05	0.01	−0.08	0.03	0.03	0.01	0.02	0.01	0.04	−0.01	−0.02	−0.01
DWT12		−0.01	−0.02		−0.01	−0.01				0.01	−0.01	
WL1				−0.01	−0.01		−0.01	0.01	0.01	0.01	0.01	0.01
BL1		0.06	0.01	0.08	0.03		0.09	0.05	0.02	−0.02	0.09	0.01
DWT13	−0.01			−0.01			−0.01			0.01	0.01	0.01
WW1		−0.01	−0.01		−0.01	−0.01	0.01	−0.01				0.02
BW1	0.05		−0.07	−0.01	0.04	0.02		0.03	−0.02	0.01	0.04	−0.02
DT2-T1	−0.01	−0.02							−0.01	0.02	0.01	0.03
WM2		0.01	−0.01			−0.01	−0.02	−0.01		−0.01	0.02	−0.06
BM2	0.02	0.04	0.01	0.04	0.06	0.03	0.03	0.02	0.01	0.01	0.04	0.07
DWT21		−0.01				−0.01	−0.01	−0.01			0.03	−0.02
WP2	−0.01	−0.01	0.03	0.01		0.03	0.02	0.01	−0.02	−0.01		
BP2	0.01	0.07	0.04	0.04	0.02	0.05	0.04	0.06	0.08	0.05	−0.01	−0.05
DWT22		−0.02	0.02			0.02	0.01	−0.01	−0.01	−0.01	0.03	−0.02
WL2	−0.01		−0.02	0.01	0.01		−0.02	0.01	0.01	0.01	−0.02	0.05
BL2	0.04	0.05	0.03	−0.02		0.08	0.07	−0.01	0.05	0.05	0.01	0.02
DWT23	−0.01	−0.01		0.01	0.01	0.01	−0.01		−0.01		0.01	0.02
WW2			0.01	−0.01	−0.01	−0.02		−0.01		−0.02	−0.05	−0.01
BW2	0.03		0.01	0.09	0.04	0.04	0.03	0.06	0.06	0.05	−0.03	0.08
DT3-T2			0.01					−0.01			−0.04	0.02
WM3	−0.01		−0.02	0.01	−0.01	−0.01	−0.01		−0.01			0.01
BM3	0.02	0.01	0.06	0.07	0.08	0.02	0.08	0.07	0.08	0.04	0.03	0.08
DWT31		0.01		0.02		−0.01	−0.01	−0.01			−0.05	0.03
WP3	−0.01	−0.02	−0.01	−0.02		0.01		0.01		0.01		−0.04
BP3	0.01	0.08	0.08	0.03	0.04	0.09	0.08	0.02	0.07	0.07	−0.01	0.01
DWT32	0.01	−0.01	−0.01		0.01		−0.01	0.01			−0.05	−0.02
WL3			−0.01					−0.01	−0.02		0.04	0.05
BL3	0.01	0.01	0.06		0.02	0.06	0.04	0.03	0.01	0.05	0.01	−0.05
DWT33			−0.02				−0.01		−0.02	0.01		0.04
WW3	0.01	−0.01	0.02	−0.01	−0.01			−0.01	0.01	−0.01	−0.01	−0.03
BW3	0.02	0.01	0.09	0.01	0.01	−0.01	0.05	0.04	−0.02	−0.04	0.01	0.04
DT4-T3						−0.01	−0.01			−0.01		
WM4	0.03	0.01	−0.05	−0.01	−0.01		0.01		0.01		0.03	
BM4		0.02	0.03	0.04	0.06	0.04		0.01	0.08	0.06	0.04	0.01
DWT41		0.01	−0.05	−0.01	−0.01	0.01	0.01	0.01	0.01	0.01	0.02	−0.01
WP4		0.03	0.04	−0.02		−0.01	−0.02	−0.01				−0.03
BP4			0.01	0.08	0.01	0.01	0.08	0.08	0.01	0.02	−0.04	0.03
DWT42			−0.01	−0.01	−0.01				0.01	0.01	0.03	−0.01
WL4			0.03		0.01			0.01			−0.03	0.02
BL4				0.02	0.01	0.01	0.08	0.06	−0.01	0.01	0.01	0.01
DWT43				−0.01				0.01	0.01	0.02		0.02
WW4				0.05	0.01			−0.01	−0.01	−0.02	−0.01	−0.01
BW4					0.02	0.08	0.07	0.09	0.04	−0.04	−0.07	−0.03
DT5-T4									0.01	−0.01	−0.01	
WM5					0.04	0.09			0.01	0.01		0.02
BM5						0.01	0.02	0.04	0.08	0.06	0.03	0.03
DWT51						0.09	−0.01		0.02		−0.01	0.02
WP5						−0.06	0.03	−0.01	−0.03	−0.02	−0.01	−0.05
BM5							0.07	0.08	0.05	0.02	0.03	0.05
DWT52							0.01				−0.02	−0.03
WL5							0.01	−0.01	0.02		0.01	
BL5								0.03	0.08	0.01	0.05	0.01
DWT53								−0.01	0.03			−0.04
WW5								0.07	−0.02			0.02
BW5									0.02	0.08	0.01	0.07
DT6-T5											−0.01	−0.01
WM6									0.05		0.02	0.01
BM6										0.02	0.01	0.01
DWT61										−0.01	0.01	
WP6										0.04	0.01	0.01
BP6											0.01	0.02
DWT62											0.01	0.03
WL6												0.01
BL6												0.02
DWT63												0.04
WW6												0.02
Intercept	0.02	0.03	0.07	0.05	0.03	0.02	0.04	0.01	0.05	0.04	0.05	0.14
Adjusted R^2^	53.98	47.73	51.48	50.88	48.92	46.22	31.43	59.05	61.4	57.96	24.19	49.67

BM, BP, BL, BW indicate body condition score prior to pre-breeding, at pregnancy diagnosis, prior to lambing, and at weaning, respectively. WM, WP, WL, WW indicate liveweight prior to pre-breeding, at pregnancy diagnosis, prior to lambing, and at weaning, respectively. DWT, DW-T indicate liveweight change within age group and between age groups, respectively. Blank space indicates coefficient non-significant at *p* < 0.05. Model example for BCS estimation (e.g., BM4 = 0.02 + 0.06 BM1+ … +0.03 WM4, adj. R^2^ = 54%).
